# Complete Genome Sequence of VpKK5, a Novel Vibrio parahaemolyticus Lytic Siphophage

**DOI:** 10.1128/genomeA.01381-14

**Published:** 2015-01-08

**Authors:** Tamrin M. Lal, Julian Ransangan

**Affiliations:** Borneo Marine Research Institute, Universiti Malaysia Sabah, Jalan UMS, Kota Kinabalu, Sabah, Malaysia

## Abstract

This paper describes the complete sequence of a novel lytic marine siphophage, VpKK5, that is specific to Vibrio parahemolyticus.

## GENOME ANNOUNCEMENT

Vibrio parahaemolyticus is an emergence of bacterial pathogens implicated in fish vibriosis (1, 2). Here, we describe the complete sequence of the V. parahaemolyticus-specific phage designated VpKK5.

The VpKK5 siphophage was isolated from coastal sand sediment of Sabah, Malaysia. The genome was extracted and purified using the DNeasy blood and tissue kit (Qiagen) according to the manufacturer’s instructions. A DNA library was prepared using Nextera XT (Illumina) and sequenced using NGS Illumina Miseq PE sequencing (AITBiotech, Singapore). A set of reads (2 × 250,000 samples) with an average read size of 250 bp were *de novo* assembled using Velvet 1.1 (Zerbino, European Bioinformatics Institute, United Kingdom) into a single contig. The genome terminal was predicted using a tandem repeat finder (3). The complete genome sequence was then subjected to BLASTn. The open reading frames (ORFs) of the genome were predicted using three bioinformatics programs, GeneMarkS (4), GLIMMER v3.02 (5), and ORF Finder (6). The function of each ORF was predicted using the PSI-BLAST (6), ScanProsite (7), Pfam (8), InterPro (9), and NCBI Conserved Domain databases (6). The sequences of tRNAs were predicted using the tRNAscan-SE program (10). The virulence factor was analyzed against VFDB (11) and MvirDB (12) databases.

The sequencing analysis revealed that the complete genome of VpKK5 is 56,637 bp in length and has a 51.32% G+C content. It consists of 80 predicted coding sequences (CDSs) with no tRNA detected. The 80 CDSs represent 90.66% of the total genome. The genes varied from 138 bp (orf47) to 3,171 bp (orf39). Thirty-seven CDSs were hypothetically novel while the others 43 CDSs showed homology but at low identity (<62%). The protein function analyses showed some CDSs are related to the DNA replication and packaging (orf15, orf19, orf24, orf34, orf35, orf60, orf63), head structure (orf45, orf56 and orf58), tail structure (orf39, orf40, orf41, orf42, orf43), and phage-bacteria interaction property (orf62). Interestingly, the genome sequence of the VpKK5 did not exhibit homology to any virulence factors. Unfortunately, the genome end cannot be determined in this study, but the deposited VpKK5 genome was arranged from replication to structural genes.

The study concluded that the genome of the *Vibrio* phage VpKK5 is novel. Lack of virulence factors would allow the phage to be used in phage therapy. The future applications of this novel phage are promising, particularly in therapy against V. parahemolyticus infection.

### Nucleotide sequence accession number.

The complete sequence of the VpKK5 genome was deposited in GenBank under the accession no. KM378617.
